# Does lifestyle intervention lower clinically significant cognitive impairment risk?

**DOI:** 10.1002/alz.71668

**Published:** 2026-07-09

**Authors:** Mark A. Espeland, Rebecca H. Neiberg, Stephen R. Rapp, José A. Luchsinger, Bonnie C. Sachs, Michelle M. Mielke, Owen Carmichael, Sevil Yasar, Haiying Chen, Adam P. Spira, Chinedu T. Momoh, Laura D. Baker, Denise K. Houston, Lynne M. Wagenknecht, Charles Semelka, Tara Beckner, Kathleen M. Hayden

**Affiliations:** ^1^ Department of Internal Medicine Wake Forest University School of Medicine Winston‐Salem North Carolina USA; ^2^ Department of Biostatistics and Data Science Wake Forest University School of Medicine Winston‐Salem North Carolina USA; ^3^ Department of Social Sciences and Health Policy Wake Forest University School of Medicine Winston‐Salem North Carolina USA; ^4^ Department of Psychiatry and Behavioral Medicine Wake Forest University School of Medicine Winson‐Salem North Carolina USA; ^5^ Departments of Medicine and Epidemiology Columbia University Irving Medical Center New York New York USA; ^6^ Department of Neurology Wake Forest University School of Medicine Winston‐Salem North Carolina USA; ^7^ Department of Epidemiology and Prevention Wake Forest University School of Medicine Winston‐Salem North Carolina USA; ^8^ Pennington Biomedical Research Center Baton Rouge Louisiana USA; ^9^ Department of Medicine Johns Hopkins School of Medicine Baltimore Maryland USA; ^10^ Department of Mental Health Department of Psychiatry and Behavioral Sciences Johns Hopkins Bloomberg School of Public Health Johns Hopkins School of Medicine Johns Hopkins Center on Aging and Health Baltimore Maryland USA; ^11^ Division of Public Health Sciences Wake Forest University School of Medicine Winston‐Salem North Carolina USA

**Keywords:** behavioral intervention, cognitive health, prevention

## Abstract

**INTRODUCTION:**

Multidomain lifestyle interventions have been demonstrated to benefit cognitive function but it is unknown whether they reduce risk for clinically significant cognitive impairment sCI (mild cognitive impairment or dementia).

**METHODS:**

The Action for Health in Diabetes administered repeated cognitive assessments in adults who had been enrolled in a 10‐year clinical trial of a multidomain Intensive Lifestyle Intervention (ILI) versus Diabetes Support and Education (DSE).

**RESULTS:**

*N* = 3655 underwent at least one assessment of cognitive status during 12–14 years of post‐trial follow‐up. The relative impact of ILI on sCI incidence varied by baseline body mass index (BMI, interaction *p* = 0.004): hazard ratio (HR) = 0.64 [0.46,0.89] for BMI 25–29 kg/m^2^, HR = 0.98 [0.85,1.16] for BMI 30–39 kg/m^2^, and HR = 1.40 [1.01,1.93] for BMI ≥ 40 kg/m^2^.

**DISCUSSION:**

A long‐term multidomain lifestyle intervention may markedly reduce the risk of incident sCI among adults with type 2 diabetes and overweight but not obesity.

## Highlights

- The cognitive status of adults with type 2 diabetes was repeatedly assessed.
- They had been enrolled in a 10‐year randomized controlled trial (RCT) of a multidomain weight loss intervention.
- Among those with baseline overweight, incident cognitive impairment was reduced.
- Among those with baseline obesity, incident cognitive impairment was not reduced.
- Among those with the greatest obesity, cognitive impairment was increased.

## 1 BACKGROUND

There is emerging evidence from some^1^, ^2^, ^3^, ^4^ but not all^5^, ^6^ well‐designed clinical trials that multidomain lifestyle interventions, that is, interventions that target improvements in multiple domains such as nutrition, physical activity, and health monitoring, may slow cognitive decline. It has not been established whether they may also reduce the risk for clinically significant cognitive impairment (sCI), usually defined as mild cognitive impairment and dementia.

We previously reported that the Action for Health in Diabetes (Look AHEAD) randomized controlled clinical trial of a 10‐year multidomain intensive lifestyle intervention (ILI) targeting caloric restriction, increased physical activity, improved nutrition, and cardiometabolic risk factor monitoring found no reduction in prevalence of sCI relative to a comparator of diabetes support and education (DSE).^7^ This initial report was based on a single cross‐sectional assessment 10‐13 years from randomization and, thus, did not capture the accrual of cases over an extended span of time.

This null finding for sCI perpetuated across additional analyses, with no overall findings of benefits for cognitive function both proximal to the cessation of the intervention^8^ and several years later.^9^ Across these analyses, however, there was evidence that the multidomain lifestyle intervention may have provided benefits for individuals who initially did not have obesity, that is, their baseline body mass index (BMI) was < 30 kg/m^2^ (to enroll, individuals were required to have body mass index (BMI) ≥ 25 kg/m^2^) but not for individuals with obesity based on pre‐specified subgroup analyses. Odds ratios (ORs) [95% confidence intervals {CIs}] for sCI ranged from OR = 0.70 [0.40,1.22] for individuals with baseline BMI 25–29 kg/m^2^, OR = 1.04 [0.70,1.39] for those with BMI 30–39 kg/m^2^, and OR = 1.46 [0.83,2.56] for individuals with BMI ≥ 40 kg/m^2^.^7^ While all these confidence intervals included 1.0 and, thus, do not rule out benefit or harm, the interaction between intervention assignment and BMI group reached statistical significance (*p* = 0.03).

This interaction was also apparent for cognitive function. In the subset of 978 participants enrolled in the Look AHEAD Movement and Memory Ancillary Study who were assessed an average of 8 years post‐randomization, among those who had BMI 25‐29 kg/m^2^ at baseline, ILI was associated with a mean benefit for composite cognitive function (z‐score) of 0.276 [0.033,0.520] standard deviation (SD) compared with a deficit of −0.086 [−0.194,0.021] SD among those with BMI ≥ 30 kg/m^2^ (interaction *p* = 0.008).^8^ When the full cohort underwent cognitive testing an average of 10–13 years post‐randomization, composite cognitive function averaged 0.047 [−0.086,0.179] SD better for ILI versus DSE among those with baseline BMI 25–29 kg/m^2^, −0.007 [−0.073,0.059] worse among those with BMI 30–39 kg/m^2^, and −0.091 [‐0.202,0.020] worse among those with BMI ≥ 40 kg/m2 (interaction *p* = 0.03).^9^

As we describe below, the follow‐up of the Look AHEAD has continued for an additional 12–14 years post‐trial to observe more cases of adjudicated cognitive impairment. In this manuscript, we assess whether there is evidence for benefit from the intervention that has emerged during this additional follow‐up. Other trials have found that important outcomes may only emerge as “legacy effects” years after the cessation of the intervention.^10^, ^11^ We have two main research goals. First, we extend our original cross‐sectional analyses that compared rates of sCI between intervention groups to include the cases that have accumulated during the additional years of follow‐up. Second, building on the earlier cross‐sectional findings described above, we use the additional cases to assess whether the intervention may only be effective in a subset of individuals based on an individual's initial obesity status. Our guiding hypothesis is that benefits of ILI with respect to sCI may be limited to individuals who are initially overweight. In supporting analyses, we also examine whether there may be different windows for benefit based on an individual's age or a marker of age‐related health status (a deficit accumulation frailty index), and also by sex and a marker of genetic risk (apolipoprotein E *[APOE]* ε4 carriage). We also explore the role that differences in weight loss trajectories may have in our findings.

## 2 METHODS

**RESEARCH IN CONTEXT**

1. **Systematic review**: Based on our review of the literature, multidomain lifestyle interventions that target multiple health‐related behaviors may slow or prevent cognitive decline across several years among at risk individuals. It is unknown whether such interventions may reduce the incidence of clinically significant cognitive impairment.
2. **Interpretation**: Our findings suggest that a multidomain lifestyle intervention targeting weight loss results in a marked reduction in the incidence of clinically significant cognitive impairment among adults with type 2 diabetes who are overweight but are not obese. Among such individuals with high levels of obesity, the intervention may accelerate the incidence of cognitive impairment.
3. **Future directions**: Our findings support the treatment of overweight in adults with type 2 diabetes to prevent cognitive impairment. Additional research is warranted to identify the underlying mechanisms for the heterogeneous intervention effects we describe.

The Look AHEAD trial enrolled 5,145 adults, aged 45–76 years, with established type 2 diabetes in 2001–2004.^12^ All participants met the following criteria: 45–76 years of age, BMI ≥ 25 kg/m^2^ (≥ 27 kg/m^2^ if on insulin), HbA1c < 97 mmol/mol (11%), systolic/diastolic blood pressure < 160 / < 100 mmHg, triglycerides < 600 mg/dl, and successful passing of a maximum graded exercise test. After the intervention phase of the trial ended September 11, 2012, *N* = 3,986 (77.5%) of its current participants were enrolled in a clinic‐based continuation study with follow‐up from 2013 to 2016. Subsequently, 3,661 (92%) of the remaining cohort entered a clinic‐based extension study from 2016 to 2020 and cognition was assessed once during this follow‐up. Participants were then given the opportunity to enroll in the Look AHEAD Aging study with annual telephone‐based follow‐up beginning in 2022. See Espeland et al.^13^ for details on the study timeline.

### 2.1 Standard protocol approvals, registrations, and participant consents

Informed consent was initially obtained from all volunteers. The Look AHEAD study protocol was initially approved by Institutional Review Boards at all study sites; however, this was later rescinded at one site whose participants are not included in our analyses. The current Look AHEAD Aging protocol was approved by a central Institutional Review Board at the Wake Forest University School of Medicine. Look AHEAD was registered as ClinicalTrials.gov (NCT00017953).

### 2.2 Interventions

The ILI targeted daily calorie goals of 1200–1800 according to initial weight and ≥ 175 min/week of physical activities such as brisk walking.^14^ The goal was weight loss of ≥ 7%. Intervention sessions occurred weekly over months 1–6 and then tapered to 3 per month for the remainder of the first year, and monthly thereafter with additional support with monthly phone or e‐mail contacts. The DSE intervention involved three group sessions per year on diet, physical activity, and social support.^15^ The mean [range] lengths of intervention periods for ILI and DSE participants included in the analyses for this manuscript were both 9.8 [8.4,11.1] years.

### 2.3 Baseline risk factors and subgroups

Data were collected by trained and masked staff, including weight and height to compute BMI. Demographic information was based on self‐report. Participants were given the option to report biological sex as female or male. *APOE* ε4 allele carrier status was determined for participants who provided consent.^16^ Deficit accumulation frailty was determined based on 38 components of age‐related health status based on medical histories, clinic‐based assessments, behaviors, functions, and abilities.^17^

### 2.4 Cognitive function

Centrally trained and certified staff conducted standardized assessments of cognitive function between August 2013 and December 2014 during a post‐intervention continuation of Look AHEAD follow‐up. These assessments were used to adjudicate cognitive impairment 10–13 years after the original randomization. Clinic‐based cognitive assessments for the adjudication of cognitive impairment were repeated once more during years 16–18. The cognitive battery measured attention, concentration, verbal learning and memory, working memory, other executive function abilities, processing speed, and global cognitive functioning.^8^

Data collection for the subsequent Look AHEAD Aging study cognitive outcomes was conducted annually via standardized telephone interviews.^13^ The transition from the prior clinic‐based assessments conducted in the Look AHEAD program to remote assessments was necessitated by the closing of the study's clinical sites. The cognitive battery assessed global cognitive function, memory, attention, executive function, and language using tests that could be administered via telephone.^13^ We include all cognitive assessments during the first three years of the Look AHEAD Aging program.

### 2.5 Adjudication of cognitive impairment

The adjudication of cognitive status was not conducted in the Look AHEAD study until the end of the 10‐year intervention phase of the trial. At this point, a masked panel of experts adjudicated cognitive status to identify sCI using all available data. Initially, potential cases included participants whose clinic‐administered Modified Mini‐Mental State Examination (3MS) test scores fell below pre‐specified age‐ and education‐specific cut points.^7^ During Look AHEAD Aging, these cut points were based on the Modified Telephone Interview for Cognitive Status (TICSm) scores.^13^ When participants’ scores fell below the cut points, this triggered the telephone administration of the Functional Assessment Questionnaire (FAQ) to a friend or family member identified by the participant to query functional status in instrumental activities of daily living.

Two adjudicators independently reviewed all cognitive test scores, the FAQ and medical and health information to make their primary classification (no impairment or sCI, i.e., mild cognitive impairment of probable dementia). Adjudicators used a separate classification of “Cannot classify” if they could not make a confident classification due to a variety of reasons (e.g., depression, illness, incomplete data): these were not counted as cases in our analyses.

When both adjudicators agreed on the primary classification, it was recorded as final. If they disagreed, the case was referred to the full five‐member committee for discussion until consensus was reached.

Participants continued to be followed after an initial adjudication of mild cognitive impairment and thus may have undergone subsequent adjudications: our analyses focus on the time until the first incidence of sCI, which could be either mild cognitive impairment or probable dementia. Once probable dementia was adjudicated, no further cognitive follow‐up was attempted.

### 2.6 Statistical analysis

We prepared a CONSORT‐like diagram to summarize the assessment schedule used to generate our analytical database (Exhibit SE 1). Chi‐squared and *t*‐tests were used to determine between‐group differences with respect to risk factors for sCI. Changes from baseline over time in weight between groups were plotted based on means from mixed effects models. Our primary analyses were based on proportional hazards regression to compare the incidence of sCI (i.e., the first time it was adjudicated, regardless of later adjudications) between intervention groups. We did not include covariates because the intervention groups were balanced with respect to risk factors.^7^ Supporting analyses were based on illness‐death models (sometimes referred to as semi‐competing risk models) which account for the competing risk of death, the risk of death following adjudication of sCI, and censoring due to lost follow‐up.^18^ Subgroup comparisons (obesity status, age, sex, deficit accumulation frailty, and *APOE* ε4) were based on tests of interaction within the proportional hazards models. Using mixed effects models, we also examined whether differences in weight loss and weight trajectories were related to sCI risks. Analyses were conducted using SAS/STAT 14.3 software.

## 3 RESULTS

Of the 4906 participants enrolled in the Look AHEAD trial for whom institutional review board (IRB) approval was in place, 662 (13%) had died and 599 (12%) others had refused further follow‐up or had been lost to contact prior to the first assessment of cognitive status (Table 1). Rates that individuals were assessed or censored were similar in both intervention groups. Table 1 also describes baseline characteristics used in subgroup analyses for these participants by intervention assignment and whether they contributed cognitive assessments or had been lost to follow‐up or died prior to completing these. There are only minor differences in these characteristics between intervention groups among participants who underwent cognitive assessments.

**TABLE 1:** Look AHEAD: Characteristics of participants by cognitive assessment status and intervention assignment: DSE versus ILI.

		DSE (*n *= 2455)			ILI (*n *= 2451)		
Parameter	Overall (*n *= 4906)	Censored prior to assessment	Died prior to assessment	At least one assessment	Censored prior to assessment	Died prior to assessment	At least one assessment
Overall, no =. (%)		306 (12.5)	351 (14.3)	1798 (73.2)	293 (12.0)	311 (12.7)	1847 (75.4)
Baseline BMI category, no. (%), kg/m^2^							
< 30	727 (14.8)	40 (13.1)	42 (12.0)	265 (14.7)	36 (12.3)	46 (14.8)	298 (16.1)
30–39	3088 (62.9)	198 (64.7)	224 (63.8)	1143 (63.6)	191 (65.2)	193 (62.1)	1139 (61.7)
40+	1091 (22.2)	68 (22.2)	85 (24.2)	390 (21.7)	66 (22.5)	72 (23.2)	410 (22.2)
Female sex, no. (%)							
No	2032 (41.4)	112 (36.6)	196 (55.8)	709 (39.4)	109 (37.2)	161 (51.8)	745 (40.3)
Yes	2874 (58.6)	194 (63.4)	155 (44.2)	1089 (60.6)	184 (62.8)	150 (48.2)	1102 (59.7)
Baseline age group, no. (%), years							
45–55	1482 (30.2)	110 (35.9)	42 (12.0)	568 (31.6)	91 (31.1)	64 (20.6)	607 (32.9)
56–65	2576 (52.5)	150 (49.0)	170 (48.4)	967 (53.8)	160 (54.6)	138 (44.4)	991 (53.7)
66–76	848 (17.3)	46 (15.0)	139 (39.6)	263 (14.6)	42 (14.3)	109 (35.0)	249 (13.5)
Deficit accumulation frailty, no. (%)							
1st tertile	2271 (46.3)	138 (45.1)	115 (32.8)	899 (50.1)	128 (43.8)	116 (37.3)	875 (47.4)
2nd tertile	1490 (30.4)	97 (31.7)	119 (33.9)	537 (29.9)	81 (27.7)	88 (28.3)	568 (30.8)
3rd tertile	1140 (23.3)	71 (23.2)	117 (33.3)	359 (20.0)	83 (28.4)	107 (34.4)	403 (21.8)
APOE ε4 allele carrier status, no. (%)							
No alleles	3079 (62.8)	166 (54.2)	209 (59.5)	1152 (64.1)	150 (51.2)	187 (60.1)	1215 (65.8)
1 or 2 alleles	926 (18.9)	42 (13.7)	70 (19.9)	336 (18.7)	47 (16.0)	67 (21.5)	364 (19.7)
Missing allele status	901 (18.4)	98 (32.0)	72 (20.5)	310 (17.2)	96 (32.8)	57 (18.3)	268 (14.5)

Abbreviations: APOE, apolipoprotein E; BMI, body mass index; DSE, diabetes support and education; ILI, intensive lifestyle intervention.

**FIGURE 1:**
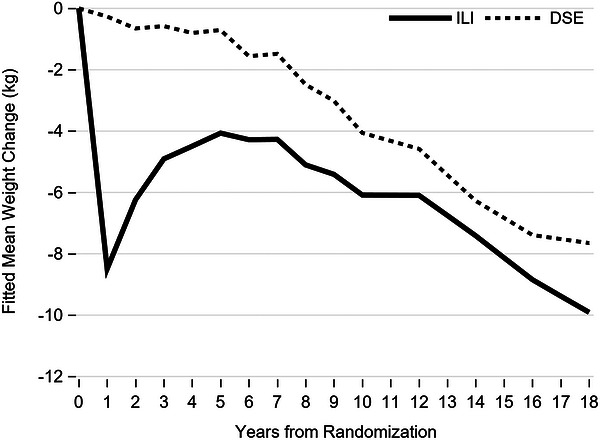
Trajectory of mean changes in weight from Look AHEAD randomization throughout follow‐up by intervention assignment: diabetes support and education (DSE) or intensive lifestyle intervention (ILI). Means were generated from mixed effects models fitted to longitudinal data collected at clinical sites.

Figure 1 portrays mean weight changes from baseline for the intervention groups through year 18 after which in‐clinic measures ceased. The largest differences between intervention groups occurred at year 1 followed by a gradual attenuation through year 5, with approximately parallel trajectories thereafter. Both intervention groups lost weight during later follow‐up, however differences between intervention groups, while diminished, were maintained.

Exhibit SE 2 tallies the cognitive status for the five waves of adjudication for participants grouped by intervention assignment, overall and for the subgroups based on baseline BMI. Also included is the mean (standard deviation) of times from randomization for cognitive assessments at each wave, which range from approximately 11 years for Look AHEAD Continuation assessments, 18 for Look AHEAD Extension assessments, and 20–22 years for the three Look AHEAD Aging assessments. *N* = 848 participants were observed to meet criteria for sCI during follow‐up.

As seen in Table 2, there were no overall differences between intervention groups with respect to incidence of sCI: hazard ratio (HR) = 0.97 [95% confidence interval 0.81,1.12], *p* = 0.70. Among ILI participants the cumulative incidence rates at years 15, 20, and 24 (end of follow‐up) were 0.091, 0.215, and 0.374; for DSE participants these were 0.092, 0.226, and 0.389 (Table 3).

**TABLE 2:** HRs [95% CIs] for clinically sCI among subgroups and by intervention assignment: ILI versus DSE.

Baseline characteristic	Clinically sCI HR [95% CI]	
	Among subgroups	ILI vs DSE
Overall *p*‐value	–	0.97 [0.85,1.12] *p *= 0.70
BMI, kg/m^2^ < 30 30–39 > 40 *p*‐Value	Reference 1.00 [0.83,1.20] 0.72 [0.57,0.90] *p *= 0.001	0.64 [0.46,0.89] 0.98 [0.83,1.16] 1.40 [1.01,1.93] Interaction *p *= 0.004
Sex Female Male *p*‐Value	Reference 1.56 [1.36,1.78] *p *< 0.001	1.00 [0.83,1.21] 0.94 [0.77,1.14] Interaction *p *= 0.59
Age 45–54 55–64 65–76 *p*‐Value	Reference 2.04 [1.69,2.46] 4.01 [3.22,5.00] *p *< 0.001	1.00 [0.71,1.39] 0.97 [0.82,1.15] 1.05 [0.80,1.38] Interaction *p *= 0.96
Deficit accumulation FI 1st tertile—lowest FI 2nd tertile 3rd tertile—greatest FI *p*‐Value	Reference 1.04 [0.89,1.22] 1.29 [1.08,1.54] *p *= 0.01	0.89 [0.73,1.08] 1.13 [0.87,1.43] 0.98 [0.73,1.30] *p *= 0.36
APOE‐ε4^*^ No alleles 1 or 2 alleles *p*‐Value	Reference 1.56 [1.33,1.83] *p *< 0.0001	0.91 [0.76,1.08] 1.03 [0.78,1.35] Interaction *p *= 0.43

* Participants with missing APOE allele status are excluded from analyses.

Abbreviations: APOE, apolipoprotein E; BMI, body mass index; CI, confidence interval; DSE, diabetes support and education; FI, frailty index; HR, hazard ratio; ILI, intensive lifestyle intervention; sCI, significant cognitive impairment.

**TABLE 3:** Cumulative incidence rates of clinically sCI by intervention group assignment from Kaplan–Meier curves: Overall and by baseline BMI.

Parameter	Follow‐up	Clinically sCI	
		DSE	ILI
Overall	< 15 years < 20 years < 24 years	0.092 0.226 0.389	0.091 0.215 0.374
BMI 25–29 kg/m^2^	< 15 years < 20 years < 24 years	0.126 0.270 0.470	0.086 0.200 0.311
BMI 30–39 kg/m^2^	< 15 years < 20 years < 24 years	0.093 0.250 0.412	0.093 0.228 0.403
BMI > 40 kg/m^2^	< 15 years < 20 years < 24 years	0.065 0.127 0.264	0.089 0.183 0.366

Abbreviations: BMI, body mass index; DSE, diabetes support and education; ILI, intensive lifestyle intervention; sCI, significant cognitive impairment.

Table 2 also describes the incidence of sCI among subgroups and whether there were relative differences in rates between intervention groups across subgroups. The overall incidence of sCI varied depending on baseline BMI (*p* < 0.001): compared with individuals with overweight, those with Class 3 obesity (BMI ≥ 40 kg/m2) had a reduced risk, HR = 0.72 [0.57,0.90]. Differences between intervention groups (ILI versus DSE) also varied by baseline obesity status (interaction *p* = 0.004) ranging from HR = 0.64 [0.46,0.89] for those with overweight, HR = 0.98 [0.85,1.16] for those with BMI from 30 to 39 kg/m2, and HR = 1.40 [1.01,1.93] for those with BMI ≥ 40 kg/m2. The cumulative incidences of sCI by years 15, 20, and 24 for participants grouped by baseline BMI and intervention assignment are listed in Table 3.

As also seen in Table 2, the incidence of sCI tended to be relatively greater among males compared to females, older versus younger participants, those with greater versus lower deficit accumulation frailty, and *APOE* ε4 allele carriers versus non‐carriers. However, within none of these subgroups was there evidence that incidence rates varied between intervention groups.

Exhibit SE 3 portrays trajectories of mean weight changes separately for participants grouped according to baseline BMI. Weight losses tended to be greatest over time among the heaviest individuals. Differences between intervention groups over time were smallest among individuals initially with overweight compared to those with obesity.

Exhibit SE 4 portrays subgroup analyses based on illness‐death models. While there are minor differences in some point estimates, the results mirror those from the proportional hazards regression. The only significant interaction is for sCI incidence across BMI subgroups.

We were interested whether the interaction between intervention assignment and BMI subgroups on the incidence of cognitive impairment might be related to differences in weight losses. Our thinking was that perhaps weight loss lowered the risk of sCI among participants with lower baseline BMI but possibly weight maintenance or weight gain lowered the risk among individuals with high baseline BMI. Exhibit SE 5 summarizes our findings. In this figure, participants are grouped according to whether they developed sCI during follow‐up. The 8‐year weight losses (8 years being the timeframe when all participants were offered interventions) for the ILI and DSE groups are portrayed. It is evident that ILI was associated with greater 8‐year weight losses than DSE for all BMI subgroups and that the heaviest participants lost the greatest amount of weight on average. It is also apparent that participants who developed sCI had slightly greater Year 8 weight losses than those who did not, except for DSE participants with baseline BMI ≥ 40 kg/m^2^ for whom weight losses were similar. Overall, however, differences between those who did and did not convert are not striking, averaging about 1 kilogram at the most, and are similar across intervention groups.

## 4 DISCUSSION

Our analysis yields three principal findings that we will discuss in turn. First, the Look AHEAD multidomain intensive lifestyle intervention had a heterogeneous impact on the risk for sCI, markedly lowering risk among individuals with overweight but providing no benefit or even increased risk among individuals with obesity. Second, the underlying mechanism(s) related to this heterogeneity appeared to be unrelated to differential amounts of weight loss or trajectories of weight losses. Third, the ILI did not appear to offer any differential benefits for cognitive impairment among individuals grouped according to age, sex, deficit accumulation frailty, and *APOE* ε4 carriage.

The Look AHEAD ILI improved many risk factors for cognitive impairment including cardiorespiratory fitness, physical activity, diabetes and blood pressure control, sleep apnea, and adiposity.^19^, ^20^ The lack of consistent benefit for sCI across the full cohort parallels the lack of overall benefit for cognitive function in the Look AHEAD cohort.^8^, ^9^ Multidomain lifestyle interventions focused on weight loss have similarly found no overall cognitive benefits in clinical trials of individuals with pre‐diabetes.^21^, ^22^ We note, however, that the J‐MINT Prime Tamba trial reported that a multidomain lifestyle intervention targeting physical exercise, cognitive training, nutritional counselling, and vascular risk management provided cognitive benefit across 18 months in a cohort aged 65–85 years that included 23% individuals with type 2 diabetes.^3^

The Look AHEAD multidomain intervention appeared to markedly reduce the long‐term risks for sCI among adults with type 2 diabetes and overweight (HR = 0.64). A potential reduction of this magnitude adds emphasis to the importance of treating overweight with lifestyle intervention as a means to protect brain health along with the many other health benefits such interventions provide.^20^

It may be difficult to replicate this finding as it may be unlikely that a similar trial will be conducted among individuals with type 2 diabetes that will have the necessary size and duration to detect weight loss intervention effects on sCI. Observational studies that explore relationships between weight loss and cognitive impairment report that weight loss often precedes dementia and co‐occurs with cognitive decline^23^, ^24^, ^25^, ^26^; however, it is expected that this largely may reflect unintentional weight loss so that relationships may be confounded and the potential benefits of weight losses might be masked. There is considerable interest whether treating overweight with incretin‐based therapies may provide cognitive benefits,^27^, ^28^ however this has not yet been established in well‐powered clinical trials.

We also report that the Look AHEAD multidomain lifestyle intervention does not increase risks for sCI among individuals with moderate levels of obesity. This supports the prescription of weight loss among these individuals for other health benefits, such as those seen in Look AHEAD.^20^

Of concern, however, is the evidence that prescription of the Look AHEAD multidomain intervention to individuals with high levels of obesity may convey cognitive harm. While there are many mechanisms through which intentional weight loss may reduce risks, it is more difficult to hypothesize why it might be harmful in the heaviest individuals. Observational studies in persons with pre‐diabetes^29^ and type 2 diabetes^30^ and in the general population^31^ suggest that higher adiposity is associated with slower cognitive decline, consistent with our findings. It is unclear whether higher adiposity is related to neuroprotective mechanisms that are reduced by intentional weight loss and if so whether this is limited to individuals with type 2 diabetes and whether it extends to other approaches to obesity treatment. This question deserves further research.

The data we present suggest that the frank differences in intervention effects between those with overweight versus those with Class 3 obesity are not directly associated with weight loss. While the heaviest participants tended to lose the most weight, there was little difference in the weight losses associated with the development of cognitive impairment in either intervention group and long‐term differences in weight losses between intervention groups were modest, that is, merely a kilogram or 2. The associations among achieved weight loss, intervention assignment, and baseline BMI were similar between participants who developed sCI compared to those who did not. Decreases in percentage body fat had little relation to cognitive function overall and across baseline BMI subgroups.^19^

The Look AHEAD ILI resulted in improved cardiorespiratory fitness, which was related to better cognitive function, however relationships were similar across BMI subgroups.^19^ Similarly, ILI lowered HbA1c levels which were related to better cognitive function,^19^, ^32^ however these associations were also similar across BMI subgroups.^19^ We have previously explored changes in cognitive function between intervention groups by weight‐related alterations in two cytokines that promote neurogenesis (leptin and vascular endothelial growth factor) but found no evidence that these were contributory and relationships did not vary by BMI.^33^ During the course of the interventions the ILI benefits for low‐density lipoprotein cholesterol (LDL‐cholesterol), triglycerides, HbA1c, and fasting glucose levels were similar among participants initially with Class 3 obesity compared to less heavy participants and were only slightly less for high‐density lipoprotein cholesterol (HDL‐C) and systolic blood pressure.^34^ These findings suggest that differential impact on cardiovascular risk does not account for the interaction we describe.

Adiposity may lead to long‐term alterations in brain energy metabolism, and this may in turn be altered by type 2 diabetes due to brain insulin resistance.^35^, ^36^ It may be that an individual's brain adapts to an environment in which this metabolism is supported by excess adipose tissue, perhaps through an increased reliance on energy sources linked to greater fat storage such as fatty acids. It is speculative, but perhaps the Look AHEAD multidomain intervention, by inducing weight loss disrupted the adapted brain energy sources among these individuals leading to less resilience to brain pathologies.

The association between weight losses and the risk of cognitive impairment may be a greater concern among older individuals. However, the Look AHEAD intervention did not appear to differentially increase risks for cognitive impairment among older versus younger individuals. This provides some support to recommend weight loss therapies to ameliorate increased risks for other conditions associated with obesity among older individuals with diabetes. In parallel, the finding that there were no differential risks associated with levels of a deficit accumulation frailty index, a loose marker of biological aging,^37^ also is encouraging and follows an earlier finding from Look AHEAD with no differential association with the outcome of cognition.^38^

Females, compared to males, in Look AHEAD had a lower prevalence of cognitive impairment spanning all age ranges^39^ and higher cognitive scores in the domains of verbal memory and processing speed.^40^ We have previously reported that this was not associated with differences in plasma estradiol or testosterone levels and appears to be an advantage unlinked to the intervention.^41^ The current finding that there were no sex‐linked differences in associations between the intervention and incidence of cognitive impairment supports this. Females (compared with males) tend to face increased risks for cognitive impairment later in life in other large cohorts^42^; it may be that obesity and or type 2 diabetes may alter this sex‐based risk differential unrelated to ILI.

The lack of a differential effect related to *APOE* ε4 carriage may suggest that any impact of the intervention on cognitive impairment is unrelated to Alzheimer's disease. This is echoed by the finding that the Look AHEAD multidomain lifestyle intervention did not result in differences in blood‐based Alzheimer's disease biomarkers.^43^

### 4.1 Limitations

Our findings are strengthened by the randomization, masking, and standardization provided by the Look AHEAD protocol. However, they may have limited generalizability in that they are drawn from volunteers meeting the eligibility requirements of the trial. sCI was not assessed until the intervention had been completed, by which time a proportion of the original participants were lost to follow‐up or had expired. The assessment schedule during follow‐up was irregularly spaced and thus the incidence of cognitive impairment was interval censored. Our illness‐death model used in supporting analyses provided some control for mortality, but other factors associated with cognitive impairment may have altered retention. Our adjudication protocols, while based on successful protocols in prior studies, are not equivalent to clinical diagnoses based on comprehensive assessment. Our decision to assess whether intervention effects vary depending on baseline obesity status was informed by earlier published results, which may influence type 2 error in our analyses, however we note that subgroup analyses based on baseline BMI were pre‐specified in the Look AHEAD protocols. Attrition (drop‐out or lost follow‐up) has the potential to bias findings, and while rates were similar between intervention groups (*p* = 0.52) and BMI strata (*p* = 0.32), biases related to unmeasured factors may have occurred. Reasons for drop‐out were not collected so that it is unknown whether drop‐out was related to incident cognitive impairment.

### 4.2 Summary

Type 2 diabetes and mid‐life obesity are well‐known to increase risks for later‐life sCI, thus finding effective strategies to reduce risks among this growing segment of the population has great public health importance. The 10‐year Look AHEAD intensive multidomain lifestyle intervention did not generally reduce risks for sCI over 20 years of follow‐up. However, it may have substantially reduced risks (hazard ratio 0.64) for sCI among those with overweight but not obesity.

## CONFLICT OF INTEREST STATEMENT

M.A.E., L.D.B., and T.B. receive research funding from the Alzheimer's Association. M.A.E receiv.es funding for serving on committees for Nestle; Annovis Bio; and Acumen Pharmaceuticals. A.P.S. has served as a paid consultant to Sequoia Neurovitality; BellSant, Inc; Amissa, Inc; and Synaptic Health, LLC. M.M.M has served on scientific advisory boards and/or has consulted for Acadia; Althira; Beckman Coulter; Biogen; Cognito Therapeutics; Eisai; Lilly; Merck; Neurogen Biomarking; Novo Nordisk; Roche; and Siemens Healthineers and receives grant support from the National Institute of Health, Department of Defense, Alzheimer's Association, and Davos Alzheimer's Collaborative. J.A.L. has received funding from Merck; Novo Nordisk; and Wolters Kluwer to attend professional meetings. O.C. has received research support from the Eli Lilly Corporation. A.P.S. has received consulting fees from Amissa, Inc; Sequoia Neurovitality; BellSantm, Inc; and Synaptic Health, LLC. Author disclosures are available in the Supporting Information.

## CONSENT

All study participants provided written, informed consent. Study partners provided written consent if questionnaires were completed in the clinic, and verbal consent if these were completed by telephone. The study was approved by the single Institutional Review Board of record at Wake Forest University School of Medicine and is conducted in accordance with the ethical standards as laid down in the 1964 Declaration of Helsinki and its later amendments or comparable ethical standards.

## Supporting information



Supporting Information

Supporting Information
