# Supplementary material for: Does lifestyle intervention lower clinically significant cognitive impairment risk?
Source: Alzheimers Dement. 2026 Jul 9;22(7):e71668. doi: 10.1002/alz.71668 (PMC13351321; doi:10.1002/alz.71668)

Supplemental Exhibit SE.1 CONSORT diagram describing the enrollment and follow-up for cognitive impairment adjudication for participants by intervention assignment: Diabetes Support and Education (DSE) and Intensive Lifestyle Intervention (ILI). Lost to follow-up (LTFU) defines participants who were unable to be contacted and/or withdrawn. The timeframes are 1) Look AHEAD Continuation (LAC-Cog) -- the first clinic based cognitive assessment of the full cohort occurring in years 10-13 post-randomization following the cessation of the intervention; 2) Look MIND ancillary study (MIND Cog) – the second clinic-based cognitive assessment of the full cohort occurring 16-18 years post-randomization; and 3) Look AHEAD Aging (LAA) -- the three annual telephone-based cognitive assessments at months 0, 12, and 24 during years 18-24 years of follow-up (depending on date of original Look AHEAD randomization).

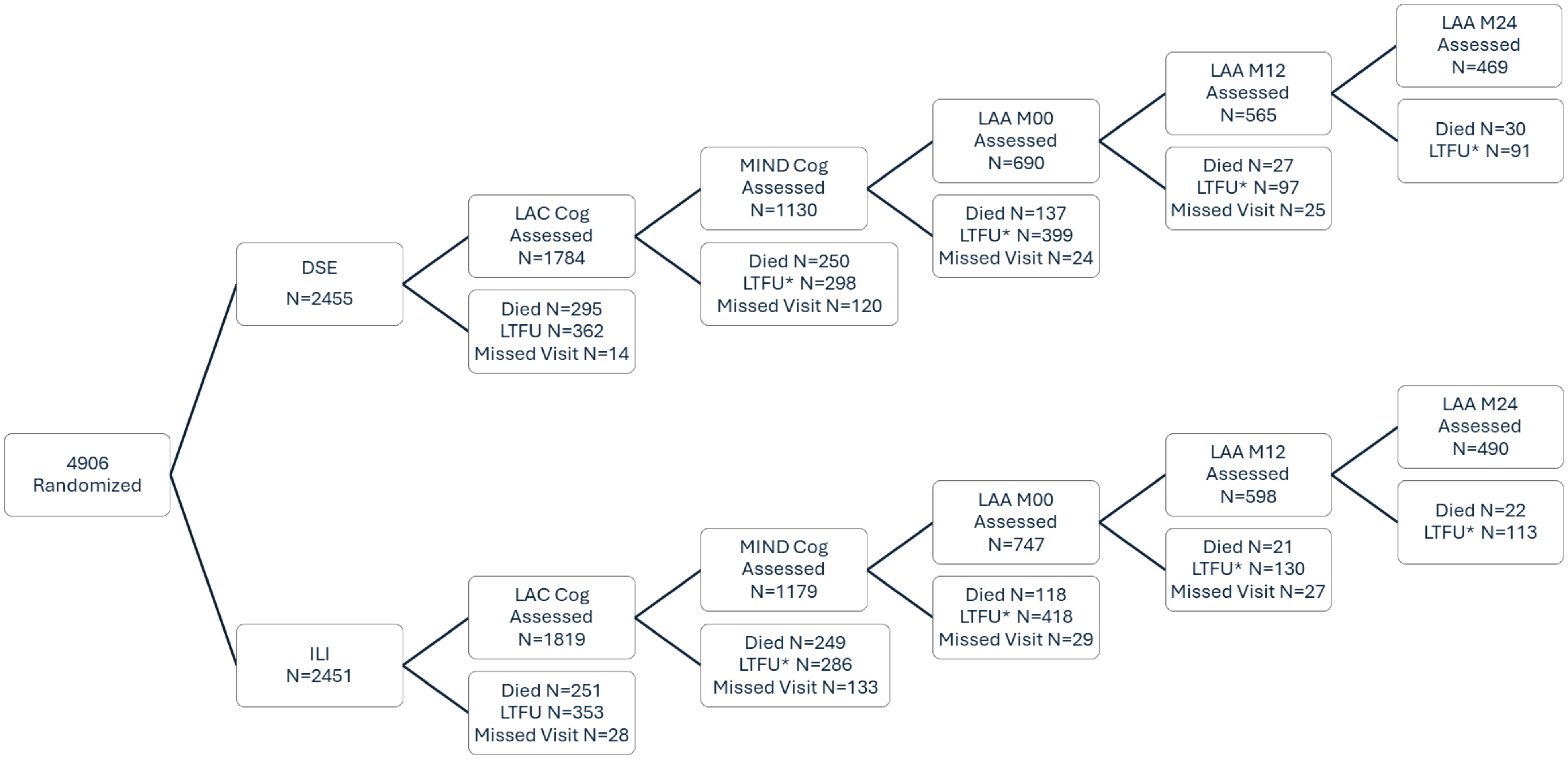
4906 Randomized
DSE N=2455
LAC Cog Assessed N=1784
Died N=295 LTFU N=362 Missed Visit N=14
MIND Cog Assessed N=1130
Died N=250 LTFU* N=298 Missed Visit N=120
LAA M00 Assessed N=690
Died N=137 LTFU* N=399 Missed Visit N=24
LAA M12 Assessed N=565
Died N=27 LTFU* N=97 Missed Visit N=25
LAA M24 Assessed N=469
Died N=30 LTFU* N=91
ILI N=2451
LAC Cog Assessed N=1819
Died N=251 LTFU N=353 Missed Visit N=28
MIND Cog Assessed N=1179
Died N=249 LTFU* N=286 Missed Visit N=133
LAA M00 Assessed N=747
Died N=118 LTFU* N=418 Missed Visit N=29
LAA M12 Assessed N=598
Died N=21 LTFU* N=130 Missed Visit N=27
LAA M24 Assessed N=490
Died N=22 LTFU* N=113

Supplemental Exhibit SE.2: Number of cognitive assessments and the prevalence of adjudicated cases of clinically significant (sCI) cognitive impairment arising from these assessments at each wave: Look AHEAD Continuation (LAC), Look AHEAD MIND (LA MIND) and, Look AHEAD Aging (three annual assessments: LAA Month 00, LAA Month 12, and LAA Month 24) by intervention assignment -- Diabetes Support and Education (DSE) and Intensive Lifestyle Intervention (ILI) -- overall and by baseline obesity status. Note that individuals continued to be followed subsequent to adjudicated sCI and thus may contribute to counts at multiple times.

	LAC		LA MIND		LAA Month 00		LAA Month 12		LAA Month 24	
	ILI	DSE	ILI	DSE	ILI	DSE	ILI	DSE	ILI	DSE
N with test	1819	1784	1179	1130	747	690	598	565	490	469
Can't Classify or Can't Classify, Functional Impairment	18	13	8	14	10	4	5	0	6	3
N with defined adjudication outcome										
Overall	1801	1771	1171	1116	737	686	593	565	484	466
Baseline BMI <30 kg/m^2	292	262	200	157	123	99	89	84	74	65
Baseline BMI 30-39 kg/m^2	1114	1120	727	713	450	439	364	357	297	292
Baseline BMI 40+ kg/m^2	395	389	244	246	164	148	140	124	113	109
Mean (±SD) time from randomization, years	11.4±0.8	11.4±0.8	17.8±0.7	17.9±0.8	19.8±0.8	19.7±0.8	20.8±0.8	20.8±0.8	21.8±0.8	21.8±0.8

**Clinically Significant Cognitive Impairment** Cases (Percent)										
Overall	163(9.1)	161(9.1)	93(7.9)	105(9.4)	146(19.8)	145(21.1)	113(19.1)	125(22.1)	82(16.9)	75(16.1)
Baseline BMI <30 kg/m^2	25(8.6)	32(12.2)	20(10.0)	15(9.6)	24(19.5)	28(28.3)	10(11.2)	24(28.6)	11(14.9)	15(23.1)
Baseline BMI 30-39 kg/m^2	103(9.2)	103(9.2)	59(8.1)	75(10.5)	95(21.1)	102(23.2)	75(20.6)	81(22.7)	54(18.2)	49(16.8)
Baseline BMI 40+ kg/m^2	35(8.9)	26(6.7)	14(5.7)	15(6.1)	27(16.5)	15(10.1)	28(20.0)	20(16.1)	17(15.0)	11(10.1)

Supplemental Exhibit SE.3 Trajectories of mean weight changes from Look AHEAD enrollment throughout follow-up by intervention assignment -- Diabetes Support and Education (DSE) and Intensive Lifestyle Intervention (ILI) -- and baseline body mass index. Means were generated from mixed effects models fitted to repeated measures.

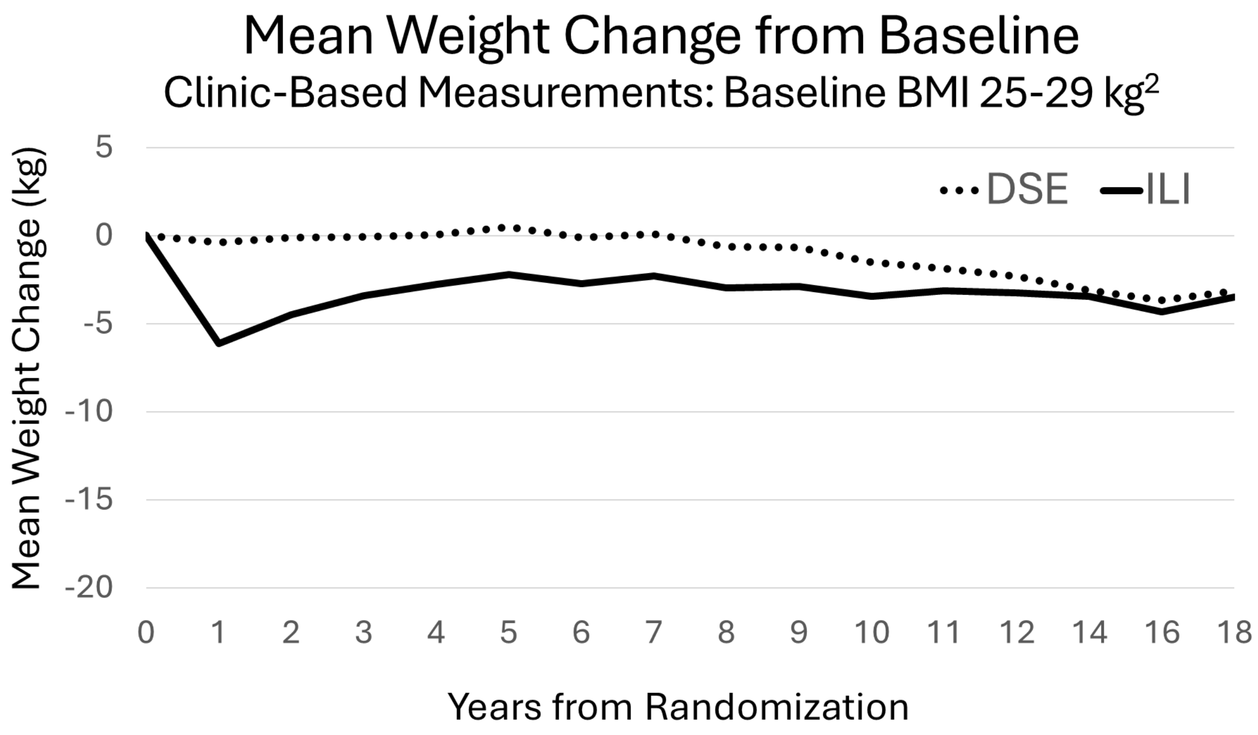

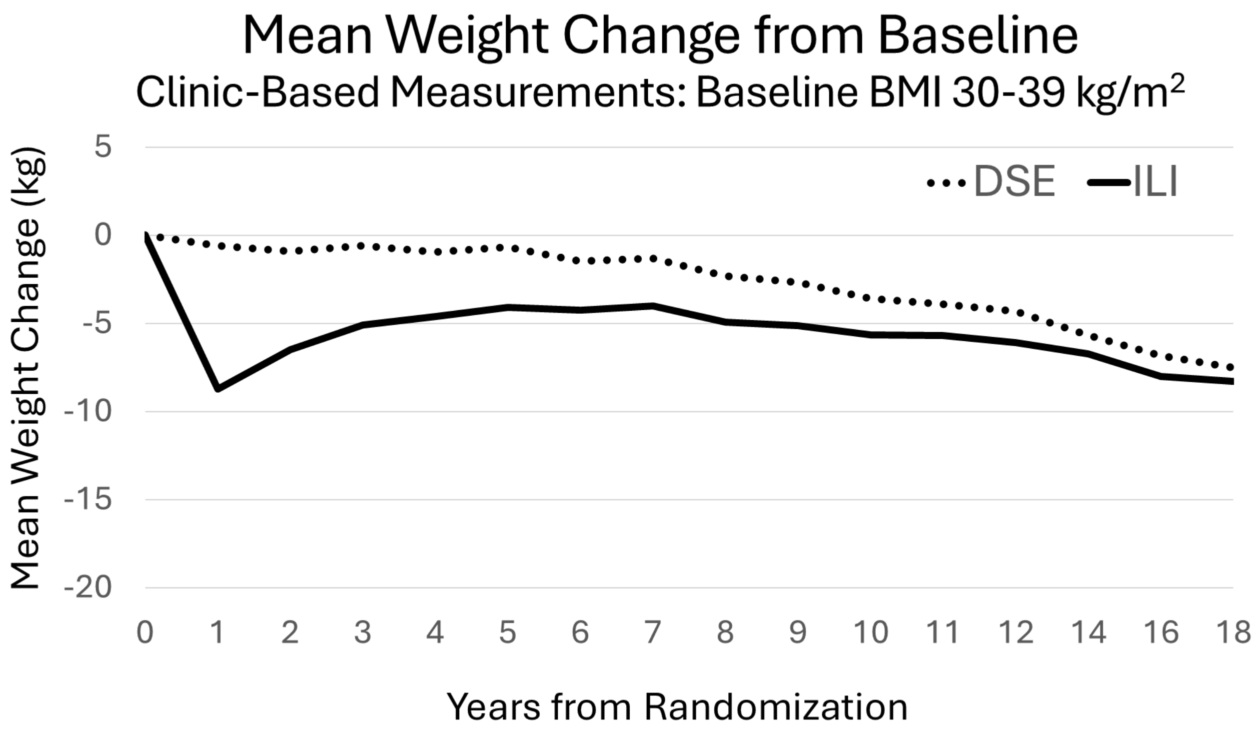
Mean Weight Change from Baseline
Clinic-Based Measurements: Baseline BMI 30-39 kg/m2
DSE
ILI
Mean Weight Change (kg)
5
0
-5
-10
-15
-20
0
1
2
3
4
5
6
7
8
9
10
11
12
14
16
18
Years from Randomization

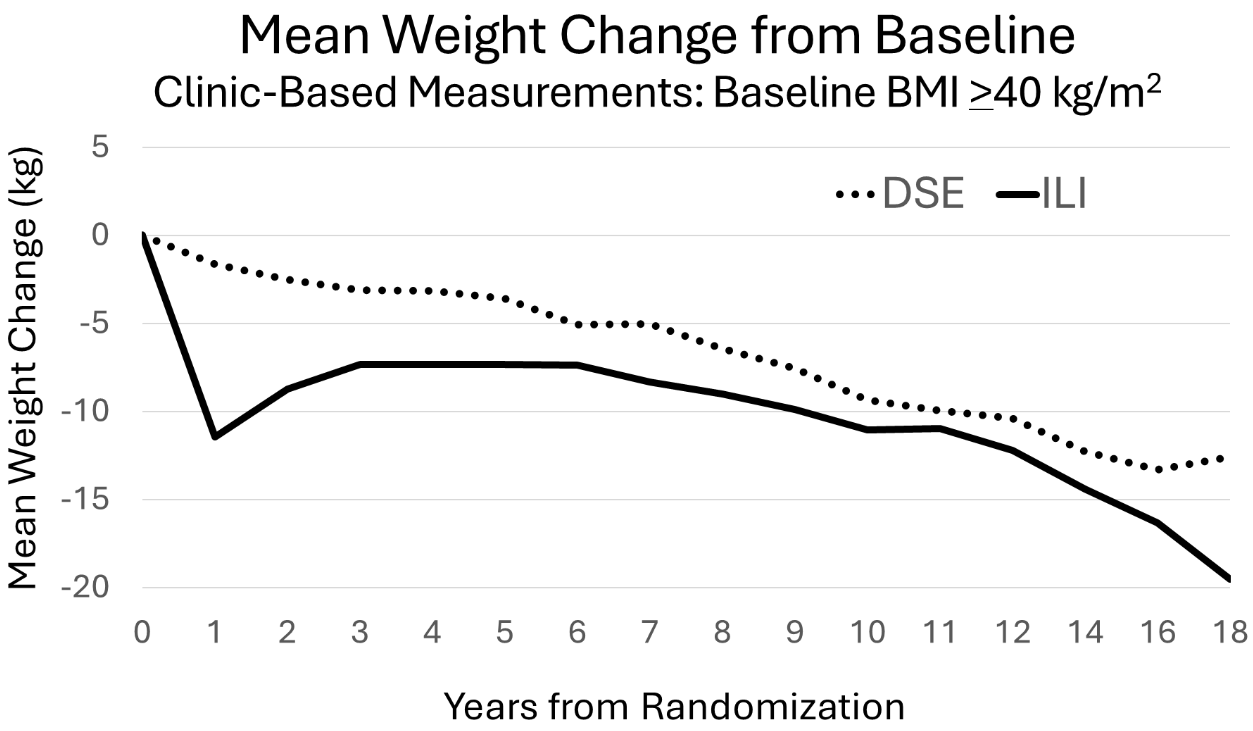
Mean Weight Change from Baseline
Clinic-Based Measurements: Baseline BMI ≥40 kg/m2
Mean Weight Change (kg)
5
0
-5
-10
-15
-20
DSE
ILI
0
1
2
3
4
5
6
7
8
9
10
11
12
14
16
18
Years from Randomization

Supplemental Exhibit SE.4. Hazard ratios for Intensive Lifestyle Intervention versus Diabetes Support and Education for clinically significant cognitive impairment: overall, for subgroups from the illness-death models.

Subgroup	Hazard Ratio [95% Confidence Ratio] for Clinically Significant Cognitive Impairment
Overall	0.95 [0.82,1.10]<br>P=0.47
Body mass index, kg/m^2	
25-29	0.59 [0.41,0.84]
30-39	0.97 [0.81,1.15]
>40	1.38 [0.97,1.94]
Interaction p-value	P=0.003
Sex	
Female	1.00 [0.82,1.22]
Male	0.89 [0.72,1.10]
Interaction p-value	P=0.43
Age	
45-54	0.91 [0.69,1.20]
55-64	1.01 [0.85,1.21]
65-76	0.99 [0.72,1.37]
Interaction p-value	P=0.81
Deficit accumulation frailty	
1st tertile	0.87 [0.71,1.07]
2nd tertile	1.07 [0.82,1.40]
3rd tertile	0.94 [0.69,1.28]
Interaction p-value	P=0.48

APOE-e4*	
No alleles	0.89 [0.73,1.07]
1 or 2 alleles	0.98 [0.73,1.33]
Interaction p-value	P=0.56

*Participants with missing APOE allele status are excluded from this analysis

Supplemental Exhibit SE.5: Mean 8-year weight losses for participants by intervention group (Diabetes Support and Education versus Intensive Lifestyle Intervention), baseline BMI, and whether they converted to cognitive impairment during follow-up. mean [95% confidence intervals] weight loss differences: converters vs nonconverters.

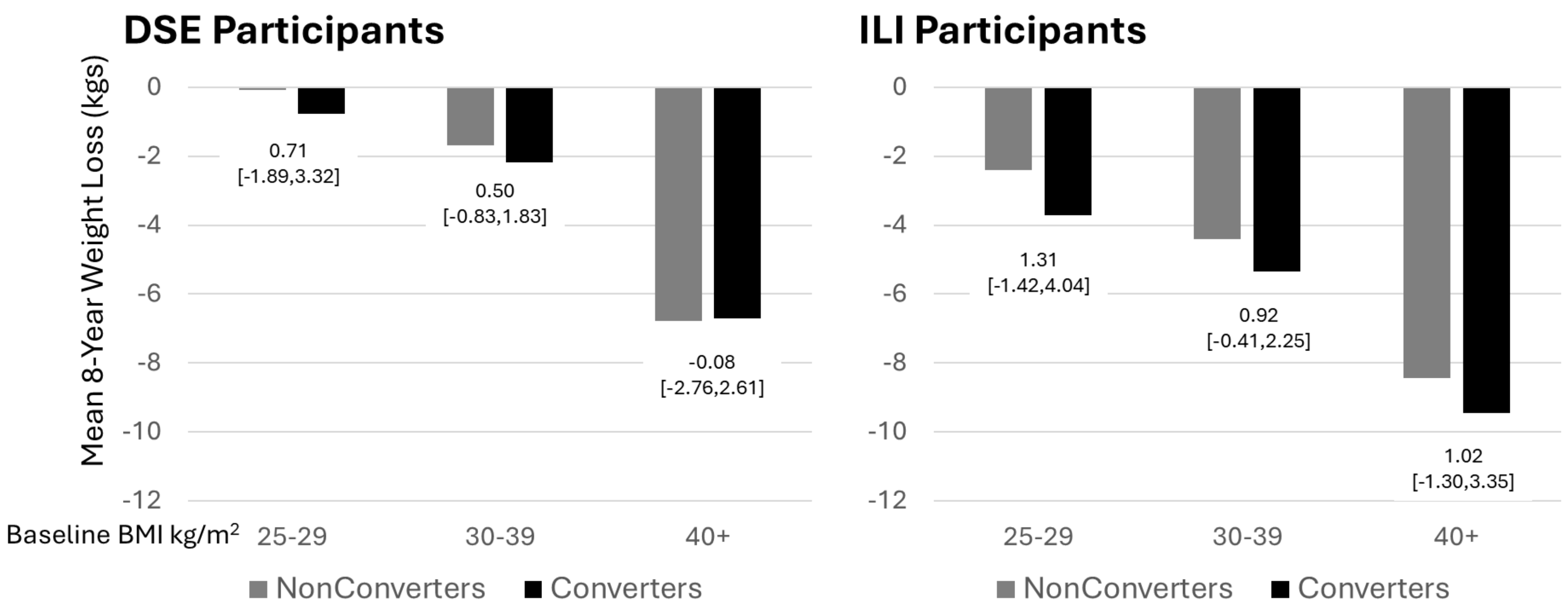

Supplement: Supplementary file 1 — Supporting Information [file ALZ-22-e71668-s001.pdf]
